# 

**Published:** 1892-04-23

**Authors:** 


### CONTENTS.

**Canon Newbolt on Suffering and Good-Will** - - - - 49

**Passing Topics.**—Pensions without Penalties - - - - 50

**The Doctor's Armchair.**—The Secret of Youth - - - - 51

**The Attitude of the Great Writers towards Disease. I.**—Sir Walter Scott - - - - - - - - 52

**Medical History from the Earliest Times. By E. T. Withington, M.A., M.B.Oxon. V.**—Medicine in Ancient Egypt 53

**Everybody's Page** - - - - - - - - - - 54

**Annotations.**—Lobworms and their Work—The New Time Coming—Lady Paget on Vegetarianism - - - - - - 55

**Notes and News** - - - - - - - - - 56

**Around the Hospitals** - - - - - - - - 57

**Scraps and Gleanings** - - - - - - - - 57

**The Practitioner's Mirror and Retrospect—**

MEDICINE.—On the Feeding of Infants - - - - - - 58

SURGERY.—On the Treatment of Nasal Polypi - - - - 58

OPHTHALMOLOGY.—Mucocele - - - - - - - - 60

PRACTITIONER'S BOOKSHELF.—The "Medical Annual" - - 60

**The Institutional Workshop—**

THE CHRONIC INDIGENCE OF OUR HOSPITALS, WITH SOME SUGGESTIONS FOR ITS REMEDY. By B. BURFORD RAWLINGS. IV.—What Might be - - - - - - - 61

FURNITURE AND FITTINGS.—A New Post-mortem Table - 63

**New York Children's Aid Society** - - - - - - - 64

**The Student of Medicine.**—Appointments—Vacancies—Pass Lists.

### EXTRA SUPPLEMENT.—THE NURSING MIRROR.

**En Passant.**—The Endowed Bed—Probationers' "Time off Duty"—"A Home for the Dying"—Lincoln Nurses—Midwives' Registration—The "House"—Progress of District Nursing xxi

**Ventilation, Disinfection, and Diet. By P. Caldwell Smith, M.D.** (*Continued*) - - - - - - - - xxii

**Male Nurses—For Reading to the Sick.**—The Vine - xxiii

**Among the Institutions — What the Register of Trained Nurses Really Is** - - - - - - xxiv

**To Relieve the Surplus Nursing Energy** - - - xxiv

**Nurses for the English in India** - - - - - - xxv

**The Nurses' Bookshelf.**—Ophthalmic Notes—How to Feed the Baby - - - - - - - - - - - - xxv

**Appointments**, xxv; **On Manners** - - - - - - xxvi

**Everybody's Opinion.**—"Talking Shop"—Holiday Experiences xxvi

**Nurses' Homes—Notes and Queries** - - - - - xxvii

**Off Duty**—A Simple Deduction—**Wants and Workers** - xxviii

